# Interrogating the “unsequenceable” genomic trinucleotide repeat disorders by long-read sequencing

**DOI:** 10.1186/s13073-017-0456-7

**Published:** 2017-07-18

**Authors:** Qian Liu, Peng Zhang, Depeng Wang, Weihong Gu, Kai Wang

**Affiliations:** 1 Institute for Genomic Medicine, Columbia University, New York, NY 10032 USA; 2 Nextomics Biosciences, Wuhan, Hubei 430000 China; 3 China-Japan Friendship Hospital, Beijing, 100029 China; 4 Department of Biomedical Informatics, Columbia University, New York, NY 10032 USA

**Keywords:** Trinucleotide repeats, Trinucleotide repeat disorders, Microsatellites, RepeatHMM, PacBio, Nanopore, Long-read sequencing

## Abstract

Microsatellite expansion, such as trinucleotide repeat expansion (TRE), is known to cause a number of genetic diseases. Sanger sequencing and next-generation short-read sequencing are unable to interrogate TRE reliably. We developed a novel algorithm called RepeatHMM to estimate repeat counts from long-read sequencing data. Evaluation on simulation data, real amplicon sequencing data on two repeat expansion disorders, and whole-genome sequencing data generated by PacBio and Oxford Nanopore technologies showed superior performance over competing approaches. We concluded that long-read sequencing coupled with RepeatHMM can estimate repeat counts on microsatellites and can interrogate the “unsequenceable” genomic trinucleotide repeat disorders.

**Electronic supplementary material:**

The online version of this article (doi:10.1186/s13073-017-0456-7) contains supplementary material, which is available to authorized users.

## Background

Trinucleotide repeat represents repetitive stretches of three base-pair motifs in DNA sequences. For example, the DNA sequence “CAG*CAG*CAG*CAG*CAG” contains five CAG repeats. Trinucleotide repeat can be located in coding and non-coding regions of the genome and is a common type of microsatellite repeats. The expansion of microsatellites, especially trinucleotide repeat expansion (TRE), has been implicated in more than 40 neurological disorders [1, 2]. For instance, the *ATXN3* gene usually contains 13–41 CAG repeats [3]; more than 55 CAG repeats in the *ATXN3* gene are pathogenic and can cause spinocerebellar ataxia type 3 (SCA3), which is a condition characterized by progressive problems with movement [4]. However, individuals with “intermediate repeat” may or may not develop SCA3. Several CAG repeat diseases are also known as polyglutamine diseases, where extensive repeats of the CAG codon result in multiple consecutive glutamines in the protein sequence. Currently, there are at least nine polyglutamine diseases, including Huntington’s disease, dentatorubropallidoluysian atrophy, spinal and bulbar muscular atrophy [5], and six types of spinocerebellar ataxia, where the repeat thresholds for pathogenicity vary in these disorders. In addition, trinucleotide expansion may also cause other types of disorders, including fragile X syndrome [6], Friedreich’s ataxia, myotonic dystrophy, and fragile XE mental retardation [2, 7]. All these genetic diseases caused by excessive expansion of trinucleotide repeats [5, 6] are collectively referred to as trinucleotide repeat disorders (TRDs).

TRDs and other disorders caused by microsatellite repeats have stimulated a large number of genetic studies. Some studies aimed to find therapeutic approaches to regulate expression level of affected genes or to shorten pathogenic repeats, for example, using zinc finger nucleases [8]. Other studies aimed to understand the molecular mechanisms contributing to repeat expansion, such as replication slippage [9–12], double-strand break repair [13, 14], base excision repair [15], and mismatch repair [16–18]. However, how these mechanisms are precisely responsible for repeat expansion is not fully elucidated yet [16].

To better understand the genotype-phenotype correlation of TRDs, it is important to detect repeat sizes accurately on personal genomes. Repeat size is critically associated with the severity of TRDs and the age of onset of TRDs symptoms. Usually, when repeat count is higher than a certain threshold, the higher the repeat count, the more severe the disorder and the earlier the onset of symptoms. The severity of TRDs may also increase from an affected ancestor generation to each successive offspring generation, demonstrating the property of genetic anticipation [19]. Therefore, precise determination of repeat counts of trinucleotide repeats will lead to an improved understanding of TRDs and the molecular mechanisms involved, and is also crucial for diagnosis, risk assessment, and prognosis of TRDs.

To determine the repeat counts of microsatellites, polymerase chain reaction (PCR) is typically used to amplify genomic regions of interest (ROIs) and then the repeat counts are determined by various techniques, such as capillary electrophoresis [20], gel electrophoresis [21], southern blot analysis [22], electrochemical detection [23], melting curve analysis [24], mass spectrometry [25], or small-molecule biosensors [26]. However, these techniques have several limitations to analyze microsatellite repeats, in that they are typically labor-intensive and time-consuming [25]. They may be difficult to be applied in high-throughput screening studies where hundreds or thousands of patients need to be genotyped at the same time. Sanger sequencing usually works for subjects with short repeats, but has substantial difficulty to infer long repeats in patients from the sequence traces, even with careful manual examination. Next-generation sequencing techniques, such as those from Illumina and Ion Torrent, have difficulty sequencing GC-rich (or GC-poor) repeat regions [27] and the repeat length in patients may easily exceed the length of the sequence reads [28]. Therefore, it is extremely difficult, if not impossible, to use these sequencing techniques to resolve longer repeats [29], sometimes referred to as “unsequenceable regions” of the human genome [28, 30].

In contrast to short-read sequencing, the development of long-read sequencing technologies, such as PacBio SMRT (single molecule real-time) sequencing [31] and Oxford Nanopore sequencing [32], enables the interrogation of more than 10,000 bp of genomic DNA sequence, thus offering the theoretical advantage to determine repeat counts in human participants [33]. However, PacBio reads have higher error rates [34, 35] (~15% on average) with a strong bias towards insertions [31]; therefore, it is not straightforward to directly use long reads to detect repeat counts, even when coupled with circular consensus sequencing (CCS), that is, sequencing the same segments many times and performing self-error correction. Similar limitations exist in the Oxford Nanopore platform, for example, a report estimated the base calling error rate of an early generation of the MinION sequencer to be 38.2% [36]. Several prior studies have explored the technical feasibility to use long-read sequencing for the analysis of TRE, such as CGG repeats in the fragile X gene [28]; however, long-read sequencing has not been routinely used in research and clinical studies of TRDs, partly due to the lack of accurate, robust, and reproducible computational tools to estimate repeat counts. More importantly, typical long-read alignment algorithms (such as BLASR [37] or BWA-MEM [38] with the “–x pacbio” parameters) may not work for reads containing long stretches of expanded repeats. One example was given in Additional file 1: Figure S1 for a patient with SCA3. This patient had a pathogenic allele with 67 CAG repeats in the *ATXN3* gene and we sequenced the repeat region and the immediate flanking region using PacBio SMRT sequencing techniques. After aligning those reads to the human reference genome, we attempted to infer the repeat counts directly from the BAM file (BAMSelf in Additional file 1: Figure S1). Clearly, this naïve method failed in finding the pathogenic allele, suggesting that more sophisticated algorithms need to be developed to address these challenges.

To improve the estimation of TRE from long-read sequencing data, we developed a novel computational tool called RepeatHMM. RepeatHMM takes a set of reads as input, uses a split-and-align strategy to improve alignments, performs error correction, and leverages a hidden Markov model (HMM) and a peak calling algorithm based on Gaussian mixture model to infer repeat counts. RepeatHMM allows users to specify error parameters of the sequencing experiments, thus automatically producing transition and emission matrices for HMM and allowing the analysis of both PacBio and Oxford Nanopore data. Below, we describe our results on evaluating RepeatHMM on simulation datasets under different sequencing scenarios and on real datasets generated from amplicon sequencing and whole-genome sequencing (WGS). It is worth noting that RepeatHMM is different from several previously published tools, such as RepeatMasker [39], tandem repeat finder (TRF) [40], and TRhist [41], which screen for simple/intersperse repeats only for a query sequence. RepeatHMM is also different from lobSTR [42], which infers microsatellites from short-read sequencing data, or PacmonsTR [43], which needs detail alignment and repeat information of every long read and uses realignment to determine repeat regions in long reads before size estimation. RepeatHMM can be accessed at https://github.com/WGLab/RepeatHMM.

## Methods

### Summary of RepeatHMM

**Fig. 1:**
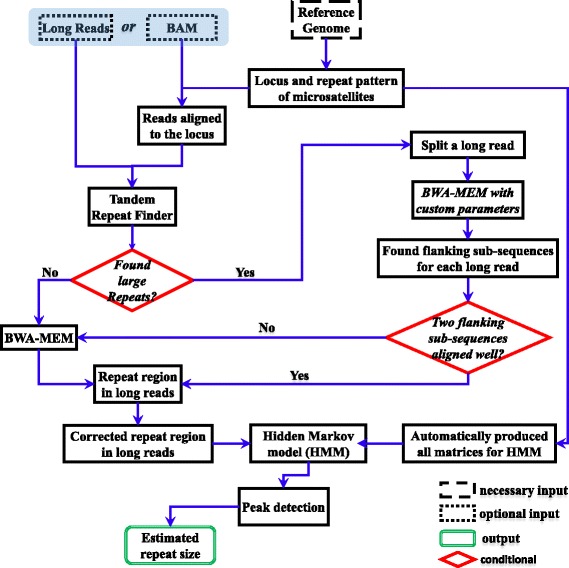
A *flowchart* of the procedure to infer repeat counts using RepeatHMM

RepeatHMM consists of several steps, as shown in Fig. 1. We used trinucleotide repeat as an example below to illustrate the procedure, but RepeatHMM can be used for microsatellites of any size.

1. Identify the location of the repeat ROI in a reference genome: first, we used a reference genome (GRCh38 was used in this study) to find the gene of interest and determined the exact start and end location of a trinucleotide repeat region.
2. The alignment of a long read: then, given a set of long reads of interests, we used two sub-processes to detect repeat regions in long reads. First, we used TRF [40] to detect repeats from a long read and then split the long read into several flanking sub-sequences and repeat regions. After that, all flanking sub-sequences were aligned to a reference genome using BWA-MEM with specialized parameters, because flanking sub-sequences still have high error rates but much shorter length. Successful alignments of ordered flanking sub-sequences were detected and used to determine the corresponding repeat regions in long reads. We called this process the split-and-align strategy. Second, all the remaining long reads, whose repeat regions were not successfully detected by the split-and-align strategy, were directly aligned with a reference genome by BWA-MEM. Those long reads were discarded if they could not be aligned to the reference genome with long flanking sequences.
3. Repeat regions in a long read: we next used long reads that covered the repeat region with upstream and downstream flanking segments for further analysis. In RepeatHMM, the minimum length of the upstream and downstream segments can be specified by users (by default, 18 bp). Furthermore, we rematched the flanking segments to a reference genome. If the alignment had high identity, we inserted several Ns between repeat regions and their flanking segments to guarantee that the flanking segments were identified as non-repeat states in RepeatHMM.
4. Error correction of a long read: to correct for sequencing errors, we used a template with perfect repeats. For example, a long read on a CTG repeat region was “CATGCTGCTGCTGGCTTCCCGCTGCTGGGTTTTTTTGTTAGTTAATGCTTTTTGCTTGCATGTCTG,” which contained a lot of insertions and deletions. To perform error correction, we designed a template with perfect CTG repeats that is 50% longer than this region and then used UnsymSeqAlg to align this read with the template, then corrected for errors based on the alignment.
5. Detection of trinucleotide repeats: each long read was used as input to a HMM [44] to estimate the repeat counts. The details on the HMM were given below. For each long read, we estimated hidden states given observed sequence and a repeat count was then estimated from the model.
6. Peak calling of repeat counts from all long reads: we next generated a histogram of all estimated repeat counts produced by HMM from all long reads. We designed a peak calling procedure described below to detect one peak (homozygous) or two peaks (heterozygous), which represented the estimated repeat counts for this participant.

### HMM for repeat detection

In this study, we used first-order HMM [44] to model the relationship between an observed sequence and a sequence of hidden states, where the probability of a state at each position only depends on the states at the previous position and the probability of an observation at each position only depends on the emission probability of the state at that position. Our HMM consists of several components, including a set of *N* hidden states *H* = {*h*_1_, *h*_2_, ⋯, *h*_*N*_}, a set of M observed symbols *S* = {*s*_1_ = *A*, *s*_2_ = *C*, *s*_3_ = *G*, *s*_4_ = *T*, *s*_5_ = *N*}, an emission matrix *E*_{*N*,5}_ = {*e*_*ij*_}_{*N*,5}_ representing the probability of *h*_*i*_ emitting *s*_*j*_, a transition matrix *T*_{*N*,*N*}_ = {*t*_*ij*_}_{*N*,*N*}_ indicating the probability of *h*_*i*_ in the previous state transiting to *h*_*j*_ in the next state, and the starting probability *P* = {*p*_1_, *p*_2_, ⋯, *p*_*N*_} giving the probability of each state before the first position of a sequence. Then, the likelihood of an observation sequence with *L* observed symbols *O* = *s*_*k*_1,_ *s*_*k*_2,_ *s*_*k*_3,⋯_, *s*_*k*_*L*_ would be *P*(*O*) = ∑_*H*_ *P*(*O*|*H*, *E*, *T*)*P*(*H*, *E*, *T*). Details on the HMM components were described below:

#### Hidden states and observed symbols

Given a microsatellite with *E* nucleotides in each repeat unit, *H* of our HMM has 3 * *E* + 1 hidden states, that is, *N* = 3 * *E* + 1: one hidden state *h*_1_ = *N* for those nucleotides which are not in microsatellites and three types of hidden states for those nucleotides in microsatellites, i.e. *h*_2_ = *r*1, *h*_3_ = *r*2, ⋯, *h*_*E* + 1_ = *re*, *h*_*E* + 2_ = *Ir*1, *h*_*E* + 3_ = *Ir*2, ⋯ *h*_2*E + 1_ = I*re*, *h*_2 * *E* + 2_ = D*r*1, *h*_2 * *E* + 3_ = D*r*2, ⋯ *h*_3*E + 1_ = D*re* indicating, respectively, the *k* th nucleotide in repeats, the insertion after the *k* th nucleotide, and the deletion of the *k* th nucleotide, where *k* ranges from 1 to *E*. Without loss of generality, take CAG repeat for example, the observed symbols *S* = {*s*_1_ = *A*, *s*_2_ = *C*, *s*_3_ = *G*, *s*_4_ = *T*} and the hidden states *H* = {*h*_1_ = *N*, *h*_2_ = *Cr*, *h*_3_ = *Ar*, *h*_4_ = *Gr*, *h*_5_ = *ICr*, *h*_6_ = *IAr*, *h*_7_ = *IGr*, *h*_8_ = *DCr*, *h*_9_ = *DAr*, *h*_10_ = *DGr*} indicating, respectively, non-repeat nucleotides, the first, second, and third nucleotide of repeats, the insertion after the first, second, and third nucleotide, and the deletion of the first, second, and third nucleotide.

#### Emission matrix

Emission matrix specifies the emission probability of a state to the four nucleotides and Ns, where each row represents a hidden state, each column represents a nucleotide, and the sum of each row is equal to 1. In an emission matrix, we considered an emission to be expected if *h*_k + 1_ emits the *k* th nucleotide acid in a microsatellite or *h*_2*E + k_ emits the (*k* + 1) th nucleotide acid and *h*_3*E + 1_ emits the first nucleotide acid. For example, for CAG repeats, we considered an emission to be expected if *h*_2_, *h*_3_, and *h*_4_ emits C, A, and G, respectively, and *h*_8_, *h*_9_, and *h*_10_ emits A, G, and C, respectively. Then, assume that a random emission rate would be 0.02 (same as the substitution error rate), then all un-expected emission probability is 0.005 (i.e. 0.02 divided by 4) and the expected emission probability is 0.985 (i.e. 1 – 0.005*3). The emission probability of an insertion state is 0.25 and of the non-repeat state is 0.2, equally for the four nucleotides or N. An example matrix of *E*_{*N*,*M*}_ for trinucleotide repeats is given in Additional file 1: Table S1.

#### Transition matrix

Transition matrix specifies the transition probabilities between different hidden states, where each row represents a state, each column represents the state to be transited to, and the sum of each row is equal to 1. Transition matrix in RepeatHMM has several specific rules based on the error profile of long reads: (1) insertion probability: the transitions from the state *rk*/I*rk*/D*r(k − 1)* to Irk, where *1 ≤ k ≤ E* and D*r(k − 1)* = D*re* if *k* = 1, indicate a possible insertion; (2) deletion probability: the transitions from *h*_1_ to D*r1* and *r*(*k* − 1)/*Ir*(*k* − 1)/*Dr*(*k* − 2) to D*rk*, where 1 ≤ k ≤ E and *r(k − 1)* = *re*, *I*r(k − 1) = I*re*, D*r(k − 2)* = D*r*(*E* − 1) if *k* = 1, indicate a possible deletion; (3) the probability to/from repeat region: *h*_1_ to *h*_2_ indicates a transition from non-repeat region to repeat region and *re*/*Ire*/*Dr*(*E* − 1) to *h*_1_ indicates a transition from repeat region to non-repeat region, and both are set to 0.02 by default; (4) the transition probability from *h*_1_ to *h*_1_ is set to 0.96 by default; (5) all non-expected transitions to *rk* or to insertion states or to deletion states have a probability close to 0, where* 1 ≤ k ≤ E*; (6) each expected transition, i.e. the transition of *r*(*k* − 1)/*Ir*(*k* − 1)/*Dr*(*k* − 2) to *rk*, where *1 ≤ k ≤ E* and *r(k − 1)* = *re*, *I*r(k − 1) = I*re*, D*r(k − 2)* = D*r*(*E* − 1) if *k* = 1, has a probability of 1 minus the sum of other probabilities in its row. Without loss of generality, take trinucleotide repeats and PacBio long reads (11% insertion rate and 2% deletion rate by default), for example: (1) insertion probability: the transitions from *h*_2_/*h*_5_/*h*_10_ to *h*_5_, *h*_3_/*h*_6_/*h*_8_ to *h*_6_ and *h*_4_/*h*_7_/*h*_9_ to *h*_7_ indicate a possible insertion and their probability is thus *i* with default value of 0.11; (2) deletion probability: the transitions from *h*_1_/*h*_4_/*h*_7_/*h*_9_ to *h*_8_, *h*_2_/*h*_5_/*h*_10_ to *h*_9_, and *h*_3_/*h*_6_/*h*_8_ to *h*_10_ indicate a possible deletion, and their probability is thus *d* with 0.02 as the default value; (3) the probability to/from repeat region: *h*_1_ to *h*_2_ indicates a transition from non-repeat region to the first nucleotide of repeats and *h*_4_/*h*_7_/*h*_9_/*h*_10_ to *h*_1_ indicates a transition from repeat region to non-repeat region, and both are set to 0.02 by default; (4) the transition probability from *h*_1_ to *h*_1_ is set to *n* with 0.96 as the default value; (5) all other non-expected transitions, including the transitions from *h*_2_/*h*_3_/*h*_5_/*h*_6_/*h*_8_/*h*_10_ to *h*_2_, *h*_3_/*h*_4_/*h*_6_/*h*_7_/*h*_8_/*h*_9_ to *h*_3_, and *h*_2_/*h*_4_/*h*_5_/*h*_7_/*h*_9_/*h*_10_ to *h*_4_, have a probability close to 0; (6) each expected transition, i.e., the transitions of *h*_4_/*h*_7_/*h*_9_ to *h*_2_, *h*_2_/*h*_5_/*h*_10_ to *h*_3_, and *h*_3_/*h*_6_/*h*_8_ to *h*_4_, have a probability of 1 minus the sum of other probabilities in its row. The example matrix of *T*_{*N*,*N*}_ for trinucleotide repeats is given in Additional file 1: Table S2. The matrix is used for all evaluations both on simulation data and real data for trinucleotide repeats in this study.

#### HMM for different trinucleotide repeats

In RepeatHMM, all trinucleotide repeat patterns have the same symbols and hidden states names and the hidden state names do not change with different repeat patterns (such as CTG or CCG). Different trinucleotide repeats have different emission matrices and *P* (Additional file 1: Table S3 for CAG repeats), but RepeatHMM can automatically readjust all matrices based on a given repeat pattern.

#### HMM for different microsatellite repeats

Different microsatellites have different repeat patterns with varying lengths and various combinations of four nucleotides. In RepeatHMM, all repeat patterns have the same symbol names (A, C, G, T, and N), but microsatellites with more nucleotides in repeat units have more hidden states. Given a microsatellite repeat pattern, RepeatHMM can automatically readjust hidden states and all matrices correspondingly. Theoretically, RepeatHMM can handle repeat patterns with any number of nucleotides in repeat units.

#### HMM for microsatellites with mixed patterns

Microsatellite repeats may contain mixed repeat patterns of the same length. For example, ATTCT repeats can be mixed with ATCCC or ATCCT or ATTCC repeats, that is, the third and fifth positions in this microsatellite repeat can be either C or T. For situations like this, the hidden states are still the same because each of mixed patterns has the same length. However, the emissions of the third and fifth positions require adjustments in the emission matrix. For example, suppose that the third position has 40% probability to be C and 60% probability to be T, then the emission probability of the third position is 0.98 * 0.4 + 0.005 for C, 0.98 * 0.6 + 0.005 for T, and 0.005 for both A and G. In such microsatellites, the mixed repeat patterns are required to be position-independent, that is, knowing the symbol in the third position does not affect the emission probability in the fifth position. RepeatHMM can handle simple mixed microsatellite repeats as described here and automatically readjust the emission matrix for repeat detections according to mixed patterns provided by users.

#### Hidden state estimation

With the matrices above, we used HMM with Viterbi algorithms [45] to estimate hidden states of each nucleotide which maximize *P*(*O*) of a given observed long read. Based on the most likely hidden states, we estimated the repeat count for the long read.

### Unsymmetrical sequence alignment and error correction

The idea of unsymmetrical sequence alignment algorithm (UnsymSeqAlg) is similar to the well-known Needleman–Wunsch algorithm or Smith–Waterman algorithm. The main difference is that UnsymSeqAlg assigns different penalties for introducing a gap in the query and the target sequence. This strategy is reasonable, because typical sequence alignment algorithms usually apply the same gap penalty for two aligned sequences and implicitly assume that two aligned sequences have the same error rates. The assumption does not hold when aligning a long read with high error rate with a template with perfect repeats (or a region in a reference genome). Thus, the penalty of a gap in long reads should be significantly larger than that in the template.

For example, suppose that the match score is *match* = 1, mismatch score is *mismatch* = –1, the gap penalty for template is *gap_in_perf* = –1 and the gap penalty for long reads is *gap_in_read* = –10, the correction of a CTG repeat region “CATGCTGCTGCTGGCTTCCCGCTGCTGGGTTTTTTTGTTAGTTAATGCTTTTTGCTTGCATGTCTG” by UnsymSeqAlg is “CTGCTGCTGCTGCTTCCGCTGCTGGTTTTTTTGTTGTTAATGCTTTTGCTGCTGCTG” where several insertions are removed. In contrast, a Smith–Waterman algorithm, with *match* = 1, *mismatch* = –1, *gap_in_perf = gap_in_read* = –1, would produce a corrected region as: “CATGCTGCTGCTGGCTTCC-C-GCTGCTGG-GTTTTTT-TGTTAGTTAATGCTTTTTGCTTGCATG-T-CTG” where more gaps (and more insertion errors) are introduced. To speed up the alignment in UnsymSeqAlg, banded alignment is also applied.

### Peak calling of repeat counts

We used the Python module scikit-learn for peak calling from the histogram of all repeat counts estimated from long reads. For microsatellites located on autosomes of a diploid genome, we assumed that the histogram was mixed by two main Gaussian models and several minor models. We then used the following steps to obtain the peak(s) from the histogram. First, we removed the repeat counts less than a minimum threshold (by default, 5) and those repeat counts with very few supporting reads (threshold specified by users). Second, we used N Gaussian components in Gaussian mixture model, where N was in the range of 3–7 (by default). For each N, we inferred the mixture model 20 times since the estimation yielded different results each time. We used Akaike information criterion (AIC) to select the best one and also required that the best mixture model was not the first or the last. The selected mixture model usually contains several individual Gaussian models. Third, we filtered the models requiring that a Gaussian model with a smaller mean should have smaller standard deviation and should have a larger amount of supporting reads. After the filtering, if there was one peak, it suggested that two alleles had the same repeat counts. If more than one peak was available, we chose one peak with the largest number of reads and identified another peak using the strategy: its supporting reads should be more than 80% (by default) of reads associated with the first peak, if its repeat count was less than the first peak; otherwise, a peak with larger count was chosen.

### Metrics for performance evaluation

To evaluate the performance, we used root mean square error (RMSE) to assess the difference between estimated repeat counts and true repeat counts. Given a set of *L* subjects each with true repeat count *RC*_*k*_ and an estimated count *PC*_*k*_,

$$ \mathrm{RMSE}=\sqrt{\frac{{\displaystyle {\sum}_{k=1}^L}{\left( R{C}_k- P{C}_k\right)}^2}{L}} $$

RMSE is a non-negative value; and the smaller the RMSE, the closer the estimated repeat counts are to the true repeat counts.

### Simulation datasets

To describe the simulation process clearly, we took the *ATN1* gene with CAG repeats as an example. Please note that the effects of PCR slippage were not considered in the simulation below.

The simulation of long reads with random start and end sites contained the following steps with user-defined parameters such as the coverage, the number of participants to be simulated, and insertion/deletion/substitution error rates:

1. Manually examined the *ATN1* gene in UCSC Genome Browser and identified the exact location of the CAG repeats. We assumed that the start position of the repeat is *start_pos*, and the end position is *end_pos*.
2. Checked the literature to obtain the minimum and maximum expansion size of both normal and pathogenic repeats and denoted the expansion limits as *min_repeat* and *max_repeat*, respectively.
3. Set *updown_size* as *max_repeat* multiplied by 25 and also set *updown_size* = 1500 bp if *updown_size* was larger than 1500; then, obtained *updown_size* bp upstream region of the repeat region and *updown_size* bp downstream region.
4. Randomly produced two counts, *c*_*i*_ and *c*_*j*_, between *min_repeat* and *max_repeat*. Two counts were produced, because each gene has two alleles, one from the father and the other from the mother. For the CAG repeat in *ATN1*, *c*_*i*_ is a random number in the range of 6–35 and *c*_*j*_ is a random number in the range of 49–88.
5. Got a random position of a CAG in repeat region of the reference genome for each count.
6. Inserted new CAG repeats at the position to produce trinucleotide repeats with *c*_*i*_ or *c*_*j*_ counts.
7. The lengths of upstream and downstream sequences were independently generated from a normal distribution with the mean of *L* bp and standard deviation of 10. *L* was set to half of *updown_size* for the smaller repeat counts and to half of *updown_size* minus half of (c_*i*_ − *c*_*j*_) × *l* for the larger repeat counts, where *l* was the length of the repeat unit.
8. Mutated the upstream sequence, the produced repeat region in (6) and downstream sequence with 11% insertion rate, 2% deletion rate, and 2% substitution rate.
9. Concatenated the mutated upstream, repeat region, and downstream to generate a random read.
10. Repeated steps (5) to (9) to generate long reads with the coverage of *cov*. Please note that *c*_*i*_ repeats had $\frac{{\mathrm{c}}_{\mathrm{j}}}{{\mathrm{c}}_{\mathrm{i}}+{\mathrm{c}}_{\mathrm{j}}}\times c o v$ long reads and *c*_*j*_ repeats had $\frac{{\mathrm{c}}_{\mathrm{i}}}{{\mathrm{c}}_{\mathrm{i}}+{\mathrm{c}}_{\mathrm{j}}}\times c o v$ long reads. That is, longer repeats had less simulated long reads and smaller repeats have more simulated long reads.
11. Repeated steps (4) to (10) to generate long reads for different participants. In the study, we simulated 100 participants.

The PCR-based simulation of long reads with fixed start and end site was similar to the random read simulation above. The only difference was that the size of the upstream and downstream sequences was determined by PCR primers rather than random simulation.

### Real datasets on patients with SCA3

Genome DNA was obtained from peripheral blood of 20 unrelated patients with SCA3 and five unaffected participants. Target sequences of the CAG repeats fragment (about 1.5 kb in *ATXN3*) were amplified using two primers (f: GATTCTCGGATTTAGGATGC; r: ATAAAGTGTGAAGGTAGCGAAC). Briefly, 50 *ng* DNA template was added to a 25 *μL* mastermix of 5 *μL* 5X PrimeSTAR GXL Buffer, 5 *mM* dNTP Mixture, 7.5 *uM* primers, 0.625 *U* PrimeSTAR GXL DNA polymerase and 15 *μL* ddH_2_O. Then, samples were amplified with an initial denaturation step of 95 °C for 5 min, followed by 35 cycles of 98 °C for 10 s, 56 °C for 15 s, 68 °C for 1 min 40 s, with a final extension step of 68 °C for 10 min, and then held at 4 °C. PCR products with equal molar ratio were barcoded, pooled, and constructed as a SMRTbell library following a standard protocol (SMRTbell Template Prep Kit 1.0). The annealed SMRTbell templates were bounded with DNA polymerase enzymes using the DNA Polymerase Binding Kit and incubated with 9 *nM* of polymerase in the presence of phospholinked nucleotides for 6 h at 30 °C. After that, the library was stored at 4 °C. Sequencing was performed within 36 h of binding. The library was sequenced on a PacBio Sequel sequencer using the manufacturer’s suggested protocols. The true repeat counts for patients were determined using capillary electrophoresis analysis. For control participants, we also sequenced the PCR products by Sanger sequencing.

### Real datasets on patients with SCA10

This dataset [46] contained PacBio long-read sequencing data on three patients with spinocerebellar ataxia type 10 (SCA10). Participants A, B, and C in this dataset had about 840, 870, and 530 repeats, respectively, as estimated by gel electrophoresis of cloned expansion fragment excised from plasmid backbone [46]. The three patients had canonical ATTCT motif mixed with other repeats and the repeat regions were in the range of 4700–6500 bp. Karen et al. sequenced *ATXN10* genes of the three participants using SMRT sequencing techniques with C2 chemistry [46].

### Real datasets on the NA12878 individual using three sequencing techniques

The subject NA12878 had been sequenced by Illumina short-read sequencing technique [47], PacBio long-read sequencing technique [48], and Oxford Nanopore long-read sequencing technique. The coverage for these three platforms were ~300X, ~50X, and ~30X, respectively. All the BAM files for Illumina short reads and PacBio long reads were downloaded from ftp://ftp-trace.ncbi.nlm.nih.gov/giab/ftp/data/NA12878/ and the BAM file for Nanopore was downloaded from https://github.com/nanopore-wgs-consortium/NA12878. The ground truth of microsatellite repeat counts is not available for NA12878, but this subject is not expected to have pathogenic alleles. We thus used the prediction from the Illumina data as the gold standard and then assessed the performance of RepeatHMM on the two other platforms.

## Results

### Overview of RepeatHMM

RepeatHMM aims at the estimation of repeat counts for a given microsatellite from long-read sequencing data. RepeatHMM can handle trinucleotide repeats as well as other more complex repeat patterns and can be used on technologies with different error profiles, such as PacBio and Oxford Nanopore. A flowchart on RepeatHMM is shown in Fig. 1 and a general overview is described below.

First, given a specific microsatellite, RepeatHMM infers the start and end position for the repeat region in a reference genome and uses a split-and-align strategy facilitated by TRF [40] and BWA-MEM [38] to find the approximate locations of the repeat region in each read. It will then use a sequence alignment algorithm UnsymSeqAlg to correct base errors in the read, to account for the differences between different types of errors, such as the much higher insertion errors (~11%) than deletion errors (~2%) in PacBio SMRT sequencing. Next, a HMM is used to estimate which nucleotides are within repeats, given the transition probabilities between hidden states and the emission probabilities from each of these states to the four observed nucleotides. The transition/emission probabilities can be directly inferred analytically rather than empirically (i.e. estimated from data) and can be automatically produced by RepeatHMM according to user-specified error profiles and repeat patterns. Finally, estimated repeat counts from all reads will be tallied and one or two peaks are inferred from these distributions, to estimate the repeat counts on each of the two homologous chromosomes.

### Estimation of repeat counts on simulation data

To evaluate the performance of RepeatHMM, we randomly simulated long reads (CAG repeats together with neighboring sequences) on the *ATN1* gene for 100 participants with varying coverages. For each participant, two alleles were simulated, including one normal allele with a repeat count sampled from 6 to 35 and one pathogenic allele with a repeat count sampled from 49 to 88. The coverage was in the range of 10–100 with a step of 10, in the range of 100–1000 with a step of 100, and in the range of 1000–5000 with a step of 1000. The read simulation used typical error models for PacBio sequencing data, with a 15% error rate, including 11% insertions, 2% deletions, and 2% substitutions (see “Methods” for details). For each coverage level, we calculated the RMSE of true repeat counts and estimated counts for these 100 participants. Additionally, we also evaluated whether the alignment itself (BAMself) could be informative for inferring repeat counts. For BAMself, we produced the BAM files using BWA-MEM with the recommended options for PacBio (i.e. − k17 − W40 − r10 − A1 − B1 − O1 − E1), and –L1 and –wG where G = Lm * 4 + hr was band width, Lm was the maximum repeat size, and hr was half of the length of the region of reads in a reference genome. Then, we determined the start and end positions of the predicted repeat region in each alignment for a long read, divided its length by 3, and rounded to the nearest integer, as the estimated repeat count for the read.

**Fig. 2:**
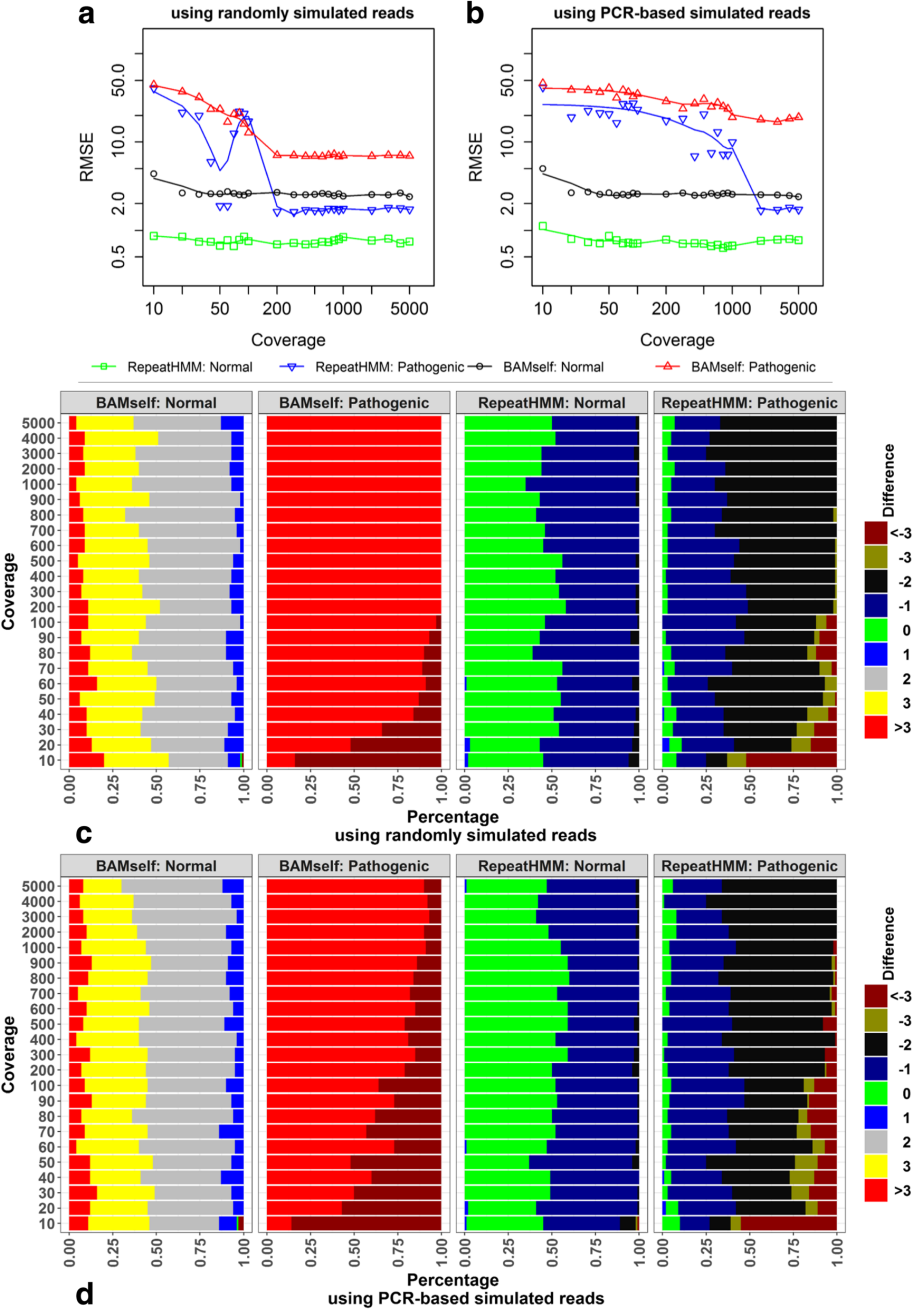
Analysis on simulation data to infer repeat counts for *ATN1*. **a** Performance on simulated long reads with random start and end sites that cover repeats. **b** Performance on simulated long reads with fixed start and end sites that cover repeats. **c**, **d** The distribution of the prediction errors (estimated repeat counts minus simulated counts) on random simulation data and PCR-based simulation data, respectively. *RMSE* root mean square error between simulated repeat counts and estimated counts for 100 participants

We found that both RepeatHMM and BAMself have improved RMSE for normal alleles when the coverage increased from 10 to 50 (Fig. 2a). When the coverage continued to increase, the RMSE of RepeatHMM levelled around 0.8, while the RMSE for BAMself levelled around 2.5. For pathogenic alleles, the RMSE of both RepeatHMM and BAMself substantially decreased when the coverage increased from 10 to 200, but RepeatHMM had much larger improvement than BAMself; when the coverage was over 200, RepeatHMM had much smaller RMSE than BAMself (1.7 versus 7). To further show the distribution of the differences between estimated repeat counts and true repeat counts, we categorized the prediction errors into several groups, including those with prediction errors less than –3, equal to –3, –2, –1, 0, 1, 2, and 3, and more than 3 (figure repeat counts and true repeat counts. Given a set oc). BAMself usually overestimated normal alleles by ≥ 2 repeats and overestimated pathogenic repeats by > 3 repeats. In comparison, RepeatHMM generated the correct repeat counts for most normal alleles with at most one repeat difference, while it underestimated by one or two repeats on pathogenic alleles when the coverage was high enough. These results suggested that the alignment itself was not able to estimate repeat counts accurately.

To further evaluate the performance of different methods on amplicon sequencing, we also generated simulation datasets under the constraints of predefined PCR primers so that all reads had similar length (not identical because of the simulation of random insertion/deletion errors). The primers for *ATN1* were designed by Primer3 [49], as CCCACCCACTACTCCCATTT (forward) and CCAGAGTTTCCGTGATGCTG (reverse). The product size in the reference genome is 762 bp. To approximate the real-world scenario of PCR, we used different amplification efficiency for the shorter and longer alleles as described in the “Methods” section. The number of participants and the categories of coverage levels had the same setting as the simulation data with random start and end sites.

Despite the use of different simulation settings, the results on PCR-based simulation data were largely consistent with the results on the simulation data with random start and end positions (Fig. 2b and d). For both RepeatHMM and BAMself, when the coverage increased from 10 to 50, the RMSE for normal alleles dropped and then levelled off. However, for pathogenic alleles, the RMSE for both algorithms continued to drop with increasing coverage. Compared with the previous simulation dataset, higher coverage of sequencing data was required to reach the same level of RMSE.

### Examination of effects when two alleles have similar repeat counts

The RepeatHMM pipeline used a peak-calling procedure to identify peaks from a histogram of repeat counts from a collection of long reads, so we next evaluated its performance when the two alleles were very similar to each other. For example, when one allele had 15 repeats and the other allele had 17 repeats, the small difference of two repeats may not be discernable by the peak-calling algorithm. To assess this, we performed additional simulation, where the count difference of two similar alleles were 1, 2, 3, 4, 5 to 6, and 7 to 9. For each count difference, the coverage was simulated from 20 (=10 * 2^1^) to 5120 (=10 * 2^9^); for each coverage level, 100 random pairs of similar alleles were simulated. The other settings of the simulation experiment were similar to the simulation described above. The RMSE of the prediction, together with the heterozygosity status of the prediction, were given in Additional file 1: Figure S2. As expected, when the two alleles were highly similar, the genotypes tended to be called homozygous. When the repeat difference increased from 1 to 4, the fraction of heterozygotes incorrectly called as homozygotes decreased from ~ 50% to ~35%, ~20%, and then ~0%, suggesting that RepeatHMM tend to over-call homozygotes when the difference between two alleles is less than 3. Overall, the RMSE was similar to that shown in Additional file 1: Figure S2, suggesting that the presence of similar alleles did not increase overall error rates but affected the calls on heterozygosity status. It is also clear that the higher coverage would help improve the prediction of heterozygosity.

### Estimation of repeat counts from real dataset on SCA3

To evaluate the performance of RepeatHMM on real data, we performed amplicon sequencing on the *ATXN3* gene on 25 participants using the PacBio Sequel sequencer. These participants consisted of 20 patients affected with Spinocerebellar ataxia type 3 (SCA3) [50, 51], with repeat counts determined by capillary electrophoresis, as well as five controls, with repeat counts determined by Sanger sequencing (Additional file 1: Table S5). SCA3 is a rare autosomal dominant disease caused by abnormally extensive duplication of CAG repeats in the *ATXN3* gene located on chromosome 14q [50, 51]. Extensive repeats in exons of *ATXN3* would affect pons and striatum, causing progressive cerebellar ataxia and even paralysis. In general, greater number of repeat counts is correlated with more severe phenotypic expression and earlier age of onset.

For the 25 participants of interest, 585,646 raw long reads with 939,895,440 bp were generated (Additional file 1: Table S4). Our sequencing experiments leveraged CCS protocol, where a CCS read is a consensus sequence generated from a multiple sequence alignment on subreads generated on a single template in a circular fashion. This set of raw data was summarized into 38,058 CCS reads with 61,063,678 bp. We therefore evaluated RepeatHMM on both raw data and the CCS data. The coverage of raw reads for most participants was more than 21,000, except sam004, sam021, sam024, and sam025, whose coverage levels were 16,988, 7750, 6915, and 10,882, respectively. In contrast, the coverage of CCS reads was more than 1300 for most participants, except sam004, sam021, sam024, and sam025, with a coverage of 1086, 569, 504, and 718, respectively.

**Fig. 3:**
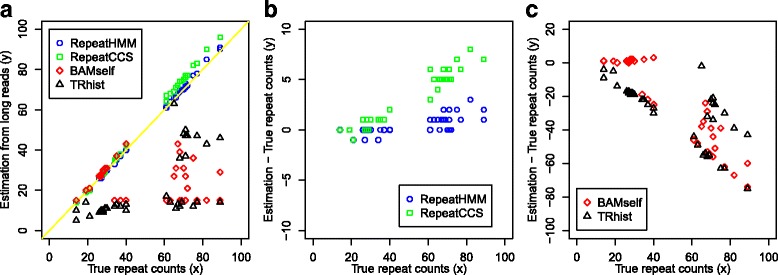
Performance of RepeatHMM, RepeatCCS, BAMself, and TRhist on estimating the repeat counts in *ATXN3* for 20 patients with SCA3 and five controls. The gold standards (*x-axis*) were determined by capillary electrophoresis for 20 patients or by Sanger sequencing for five controls. **a** *Scatterplot* of estimated repeat counts and true counts. **b**, **c** The difference of estimated repeat counts and true counts by RepeatHMM, RepeatCCS, BAMself, and TRhist. RepeatCCS refers to the use of RepeatHMM on error-corrected reads generated by the circular consensus sequencing protocol

Using the raw reads, the predicted repeat counts and the differences from the gold standards were shown in Fig. 3 and Additional file 1: Table S5. For comparison, we also run TRhist and summarized its results into repeat counts using a custom script (two repeat units were merged if their distance was less than 5 bp). RepeatHMM worked well on the raw reads (Fig. 3a and b): The difference between repeat counts determined by RepeatHMM and the gold standard was mostly 0 or 1 with only a few exceptions (Additional file 1: Table S5). The predictions for ten normal alleles in five unaffected participants and 17 normal alleles in 20 patients were identical to the gold standard, and the three normal alleles in 20 patients were underestimated by one repeat. For the pathogenic alleles of 20 patients, five predictions are identical to the gold standard, ten were overestimated by one repeat, four by two repeats, and one by three repeats. Additionally, the estimation error was largely random and was not correlated with true repeat size. In comparison, BAMself and TRhist produced very poor predictions, especially on the pathogenic alleles (Fig. 3 and Additional file 1: Tables S5 and S6).

To evaluate whether the CCS protocol could improve the predictive performance, we used RepeatHMM on CCS reads, and we referred to this analysis as RepeatCCS. The detailed results of the RepeatCCS analysis were shown in Fig. 3 and Additional file 1: Table S5. Although RepeatCCS worked better than BAMself and TRhist, it had higher error rates than RepeatHMM. Thus, in the RepeatHMM framework, CCS reads did not confer obvious advantage to raw reads in quantifying longer repeat counts.

Interestingly, we found that the prediction errors (the estimated repeat counts minus the true repeat counts) by RepeatCCS had a clear positive correlation with the true repeat counts (Fig. 3b). This indicated that the CCS protocol may be biased when assessing repeat counts and that the bias was not random. One possible reason leading to this bias may be due to the multiple sequence alignment algorithm used to generate CCS reads. When inferring consensus sequences from multiple subreads, the alignment algorithm may not be able to accurately align subreads with many repeats against each other, due to the error profile of the sequencing data. For example, given a repeat of 80 CAG triplets (240 bp), the generated data were on average ~10% longer (due to the much higher insertion rate over deletion rate), so the length of repeat regions in the CCS reads would be on average around 88 × 3 = 264 bp. RepeatHMM directly used raw reads and thus was less susceptible to this problem given appropriate adjustment of alignment parameters. Therefore, although CCS reads offered some advantages over raw reads and were preferably used in many applications (such as amplicon sequencing and RNA-sequencing), more cautions should be taken when CCS reads are used in estimation of repeat counts.

### Estimation of repeat counts from real data on *SCA10*

To further evaluate the performance of RepeatHMM on regions with more complex repeat patterns than trinucleotide repeats, we also analyzed another dataset on SCA10 [46]. The intronic region of the *ATXN10* gene contains 14 ATTCT repeats in the reference genome. However, in this dataset, many hundreds of repeat units were present in each of the patients. Furthermore, in addition to ATTCT, the repeat region also contained a small fraction of other repeat units such as ATCCT, ATTCC, and ATCCC.

**Fig. 4:**
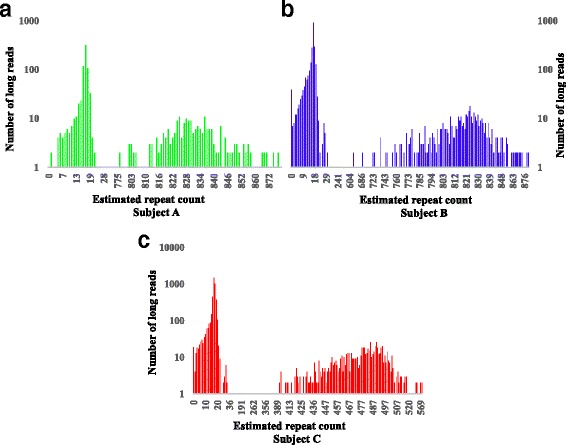
The distribution of repeat counts estimated by RepeatHMM for three patients with *SCA10*. The estimation of the pathogenic alleles by RepeatHMM for the three subjects A, B and C were 830 (**a**), 825 (**b**) and 488 (**c**), and the estimation by gel electrophoresis were ~840, ~820 and ~530, respectively

Three methods were evaluated on the raw reads in the SCA10 dataset: RepeatHMM, TRhist, and BAMself. The results were shown in Table 1 where both BAMself and TRhist failed to accurately detect the pathogenic allele in *ATXN3* in all three patients. We also noted that the consensus sequences produced by the original authors [46] gave larger estimation of repeats counts for both participants A (~30 repeats larger) and B (~64 repeats larger). In contrast, repeat sizes estimated by RepeatHMM were closer to those estimated by gel electrophoresis (Fig. 4 and Table 1) for participants A and B. For participant C, there was a larger difference between the estimation by RepeatHMM and the size inferred by gel electrophoresis, which may be because participant C contained many interrupted repeats [46]. In summary, this comparative analysis demonstrated that RepeatHMM can also work on complex repeat regions spanning thousands of base pairs with mixed repeat units.

**Table 1:** Estimation of repeat counts on the SCA10 dataset. The gel estimated counts were from the previous study on the pathogenic allele [46]

	BAMself		TRhist		RepeatHMM		Gel estimated	Estimation in [46]
Participant A	15	15	10	27	17	830	~840	~870
Participant B	15	15	5	41	17	825	~820	~884
Participant C	14	14	5	27	17	488	~530	~514

### Estimation of repeat counts from WGS data

We further evaluated RepeatHMM on WGS data on NA12878 generated on three technical platforms: PacBio SMRT sequencing (~50X coverage), Oxford Nanopore sequencing (~30X coverage), and Illumina short-read sequencing (~300X coverage). We recently generated PacBio SMRT sequencing data on a Chinese adult male (HX1) with normal karyotype (~100X coverage) [52] and we included this individual in the analysis as well. Unlike amplicon sequencing experiments, whole-genome long-read sequencing typically had much lower coverage (for example, 100X or lower), and all reads had random start and end sites that were distributed throughout the genome. We selected 15 trinucleotide repeats that were known to cause inherited neurological diseases, as well as 33 additional microsatellites with 2–5 bases as repeat units. (We determined the repeat patterns and the start/end positions of microsatellites based on the UCSC genome browser.) Since the Illumina data had high coverage (~300X) and the sizes of repeat regions are expected to be smaller than the read length (150 bp), we used the estimation of repeat counts from the Illumina data as the gold standard to evaluate the two long-read sequencing platforms.

**Fig. 5:**
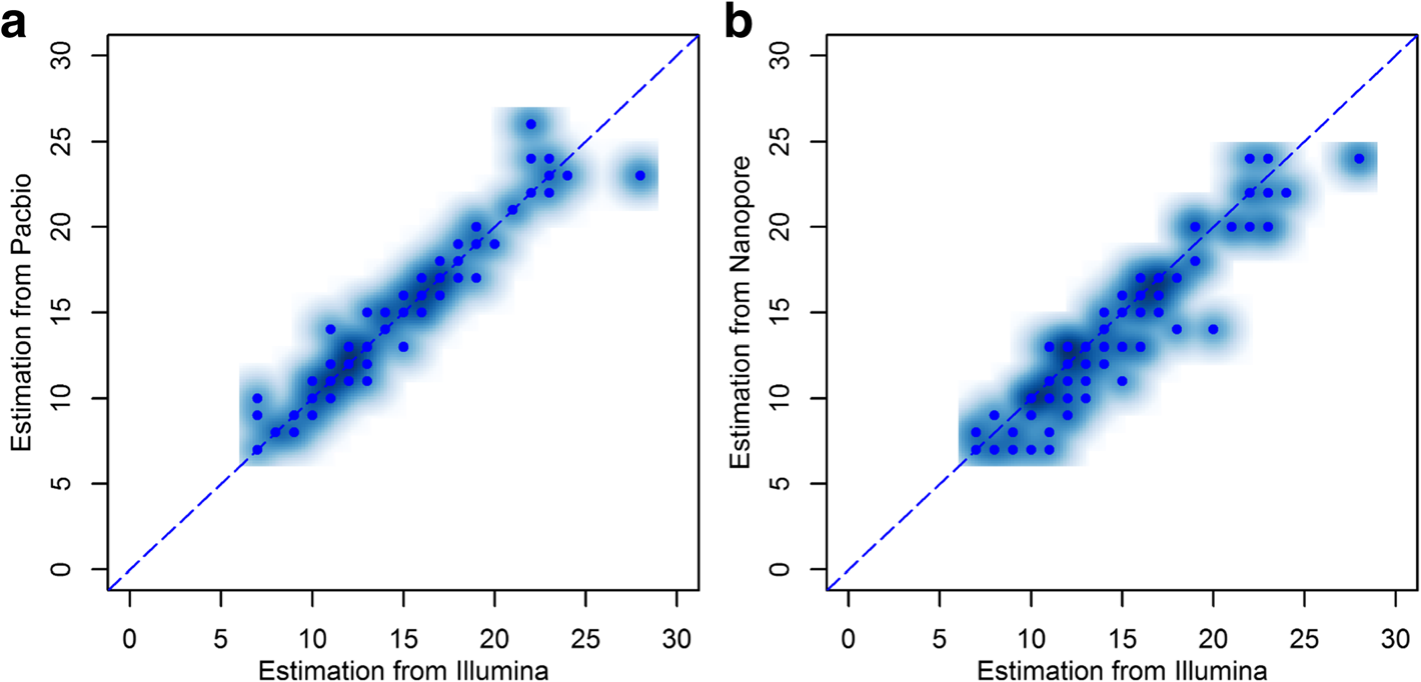
Comparison of the estimation of repeat counts on NA12878 using three sequencing platforms. The sequencing platforms include Illumina short-read sequencing, PacBio long-read sequencing (**a**), and Nanopore long-read sequencing (**b**). We examined 40 microsatellites with repeat units in the range of 2–5 bp, which are short enough to be confidently called by the Illumina data

During the analysis, for each microsatellite, we found that generally more than 80% of the short reads supported the exact repeat counts (either one or two counts) calculated by RepeatHMM, indicating that Illumina-based estimation is reliable and could be used as gold standard for comparison. The predictions from two long-read sequencing platforms were largely consistent with the gold standard (Fig. 5). Therefore, RepeatHMM could work on different sequencing platforms with different error characteristics by adjusting the model parameters. However, we also noted that there were some prediction errors, especially on predicting homozygous repeat counts (Additional file 1: Table S7). This may be due to the fact that the coverage of long-read sequencing data for NA12878 was not high enough to distinguish the alleles with similar repeat counts, as we had discussed above. In addition, some of the repeat regions cannot be confidently called by either PacBio or Nanopore data, due to the relatively low coverage. We acknowledged that this analysis focused on repeat regions with normal alleles and relatively small number of repeat units, so the results might not be extrapolated to pathogenic alleles. In summary, our exploratory analysis confirmed that RepeatHMM worked on different long-read sequencing platforms, with appropriate adjustment of model parameters in HMM.

**Fig. 6:**
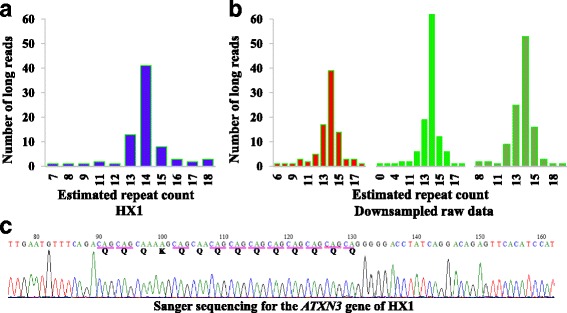
The analysis of *ATXN3* in HX1 using three different sequencing techniques. **a** Whole-genome long-read sequencing with ~100X coverage. **b** PCR-based long-read sequencing with three randomly down-sampled datasets, each with ~100X coverage. **c** Sanger sequencing. All methods concordantly predicted that there were 14 CAG repeats in *ATXN3*

We also used RepeatHMM on HX1 [52] to analyze 15 distinct types of trinucleotide repeats that were known to cause human diseases (Additional file 1: Table S7). All the repeat counts were within normal ranges, consistent with the prior knowledge that HX1 did not have a known neurological disorder. Furthermore, we analyzed the CAG repeats in the *ATXN3* gene using three different techniques: on whole-genome long-read sequencing (Fig. 6a), on PCR-based long-read sequencing (Fig. 6b), and on Sanger sequencing (Fig. 6c). Since PCR-based long-read data had high coverage, we down-sampled the dataset and produced three subsets of data, each with ~100X coverage. We found that WGS, PCR-based amplicon sequencing (three down-sampled subsets), and Sanger sequencing concordantly predicted that HX1 had 14 CAG repeats in *ATXN3*, further suggesting that RepeatHMM worked on different types of data.

## Discussion

Long stretches of trinucleotide repeats generally cannot be interrogated by Sanger sequencing or next-generation sequencing, and were traditionally regarded as “unsequenceable” genomic regions. In this study, we leveraged long-read sequencing techniques and developed a novel computational tool, RepeatHMM, to estimate repeat counts for microsatellites. Compared to existing techniques (capillary electrophoresis and southern blot) that are labor-intensive and cannot be scaled to high-throughput applications, the combination of long-read sequencing and RepeatHMM may greatly facilitate rapid and convenient estimation of repeat counts. Our results suggested that long-read sequencing has the potential to be routinely used in research studies on microsatellite repeat disorders and may be extended in clinical diagnostic applications.

RepeatHMM has several advantages over traditional approaches to determine repeat counts. First, RepeatHMM takes long reads as input and utilizes HMM for repeat region detection: HMM is computationally flexible to detect various types of repeats with different unit lengths and motifs. Although we demonstrated the usefulness of RepeatHMM on real dataset for CAG repeats and ATTCT repeats, RepeatHMM can be used for other types of trinucleotide repeats by simply specifying different sets of parameters. Second, different error profiles from various sequencers (such as PacBio sequencer and Oxford Nanopore sequencers) can also be incorporated into HMM using different parameters. Third, RepeatHMM is computationally efficient. Based on the evaluation on the SCA3 dataset, it usually took 2–12 min to analyze raw data on *ATXN3* for a participant (~21,000X coverage). The memory usage is up to 200 Mb in a 64-bit Linux machine with Python 2.7; however, please note that TRF requires more time and memory, especially if the reads contain complicated repeat patterns or if the reads are too long. Therefore, RepeatHMM is a flexible, efficient, and powerful tool to replace traditional approaches for the determination of repeat counts.

However, there are several limitations of the RepeatHMM approach. First, some repeat regions have mixed repeats, such as one CTG within many CAG, or contain interrupted repeats, such as 10 CAG repeats plus TTTTTTG followed by another 20 CAG repeats. If long reads contain non-canonical repeats, it is not straightforward to differentiate the interruption of perfect repeats from insertion/substitution errors. To address this problem, in the current version of RepeatHMM, the former scenario (several CTG within many CAG) can be formulated using simple mixed repeat patterns. For the latter case (10 CAG repeats plus TTTTTTG followed by another 20 CAG repeats), RepeatHMM would consider it as a combination of multiple single-base insertions and a deletion of a CAG and the insertions did not contribute to repeat length estimation (however, the results may be post-processed to identify stretches of continuous insertions). For even more complicated repeat patterns, the models need specialized adjustment. Second, the flanking downstream/upstream sequences of repeat regions need to be long enough for RepeatHMM to work reliably. If a long read has a short flanking sequence such as 20 bp, yet the repeat region is too long (for example, 200 repeats with more than 600 bp), few alignment software tools could correctly map the long reads to a reference genome. Thus, repeat counts in some of the long reads with short flanking sequences cannot be calculated. Third, repeat patterns and their location in a reference genome are assumed to be known a priori, that is, our method did not aim at de novo discovery of repeat patterns. Fourth, we found that RepeatHMM tended to make mistakes when two alleles have similar repeat counts, which confused the peak calling procedure. As shown in Additional file 1: Figure S2, when the size difference between two alleles was more than 2, the error rates decreased sharply. In most cases the pathogenic allele would be substantially longer than the benign allele and this problem had limited influence on the accuracy of repeat size estimation for disease analysis in practice. On the other hand, in some cases where two alleles have similar repeat counts, allele drop-out remains a critical issue and needs to be addressed in the future to avoid false negatives for the correct identification of heterozygosity. Finally, our peaking calling procedure always assumed one or two peaks in the repeat counts, which did not handle cases where extensive mosaicism is available. We may improve the peaking calling procedure in the future to address this issue.

We demonstrated a few successful examples in using RepeatHMM to quantify well-known and canonical disease-associated repeat expansions, but it is conceivable that RepeatHMM may also be useful for other microsatellite repeats, including more complicated repeats that do not always conform to canonical patterns. Microsatellites account for about 2% of human genomes with a wide distribution throughout the genome [53] and have a higher mutation rate than other regions of the genome [54]. Microsatellite repeats contribute to genetic diversity in human populations and to the development of some human diseases by affecting gene expression or the function of encoded proteins. Given the success of RepeatHMM on simple microsatellite repeats, in the future we will explore the modification of RepeatHMM to handle more complicated microsatellite repeats with mixed patterns of different lengths, as well as minisatellites with much longer repeat units (10–60 bp).

## Conclusions

In this study, we have developed RepeatHMM to detect repeat counts of microsatellites from long-read sequencing data. RepeatHMM was evaluated on both simulation data and real data and our results suggested that RepeatHMM was effective and efficient to quantify repeat counts. RepeatHMM is flexible to handle repeat patterns of any length beyond trinucleotide repeats and can incorporate different error profiles. With the wider application of long-read sequencing techniques in research and clinical settings, RepeatHMM is expected to contribute to the quantification of repeat counts and to facilitate the analysis of genotype-phenotype relationships for disease-related microsatellites.

## Additional file

Additional file 1: The matrices for the HMM, the results on estimating repeat sizes in *ATXN3* on 20 patients with SCA3 and five controls, and supplementary figures. (DOCX 353 kb)

## Abbreviations

CCS: Circular consensus sequencing
PCR: Polymerase chain reaction
RepeatCCS: RepeatHMM on Circular Consensus Sequencing reads
RMSE: Root mean square error
TRD: Trinucleotide repeat disorders
TRE: Trinucleotide repeat expansion
UnsymSeqAlg: Unsymmetrical sequence alignment
