# Supplementary material for: Interrogating the “unsequenceable” genomic trinucleotide repeat disorders by long-read sequencing
Source: Genome Med. 2017 Jul 18;9:65. doi: 10.1186/s13073-017-0456-7 (PMC5514472; doi:10.1186/s13073-017-0456-7)
Supplement: Additional file 1: — The matrices for the HMM, the results on estimating repeat sizes in ATXN3 on 20 patients with SCA3 and five controls, and supplementary figures. (DOCX 353 kb) [file 13073_2017_456_MOESM1_ESM.docx]

**Interrogating the "unsequenceable" genomic trinucleotide repeat disorders by long-read sequencing---Supplementary Materials**

## Qian Liu^1^, Peng Zhang^2^, Depeng Wang^2^, Weihong Gu^3^, Kai Wang^1,4*^

^1^ Institute for Genomic Medicine, Columbia University, New York, NY 10032, USA.

^2^ Nextomics Biosciences, Wuhan, Hubei 430000, China.

^3^ China-Japan Friendship Hospital, Beijing, China.

^4^ Department of Biomedical Informatics, Columbia University, New York, NY 10032, USA.

Additional file 1

Table S1. An example of the emission matrix from 10 hidden states (in rows) to 5 observed symbols (in columns) used in the HMM for a trinucleotide repeat. ‘*r/r'*’, ‘*e*’ and ‘*p*’ indicated random probability, error probability and correct probability, respectively. In this study, ‘*r*’=0.25, ‘*r'*’=0.2, ‘*e*’=0.005 and ‘*p*’=0.985 were used for CAG repeats.

| **10 hidden states** |  | **4 nucleotides** | | | | **N** |
| --- | --- | --- | --- | --- | --- | --- |
|  |  | **A** | **C** | **G** | **T** |  |
|  | **N** | *r* | *r* | *r* | *r* | *r* |
|  | **Cr** | *e* | *p* | *e* | *e* | *e* |
|  | **Ar** | *p* | *e* | *e* | *e* | *e* |
|  | **Gr** | *e* | *e* | *p* | *e* | *e* |
|  | **ICr** | *r'* | *r'* | *r'* | *r'* | *e* |
|  | **IAr** | *r'* | *r'* | *r'* | *r'* | *e* |
|  | **IGr** | *r'* | *r'* | *r'* | *r'* | *e* |
|  | **DCr** | *p* | *e* | *e* | *e* | *e* |
|  | **DAr** | *e* | *e* | *p* | *e* | *e* |
|  | **DGr** | *e* | *p* | *e* | *e* | *e* |

Table S2. An example of the transition matrix between 10 hidden states in HMM for a trinucleotide repeat. ‘*i*’, ‘*d*’, ‘*s*’, ‘*b*’, ‘*n*’, ‘*r*’ and ‘*p*’ indicated insert error, deletion error, average substitution error, background random error, the probability from a non-repeat region to a repeat region, the probability from a repeat region to a non-repeat region, and other transition probability among repeat regions, respectively. In this study, for PacBio data, “*b*” was set to absolute minimum larger than 0, ‘*i*’=0.11, ‘*d*’=0.02, ‘*s*’=10^-9, ‘*n*’=0.96, ‘*r*’=0.02, ‘*p*’=1-the sum of other cells in each row (0.8486 for the rows of ‘Gr’, ‘IGr’, and ‘DAr’, and 0.8686 for other rows).

|  |  | **Hidden states to be** | | | | | | | | | |
| --- | --- | --- | --- | --- | --- | --- | --- | --- | --- | --- | --- |
| **Current hidden states** |  | **N** | **Cr** | **Ar** | **Gr** | **ICr** | **IAr** | **IGr** | **DCr** | **DAr** | **DGr** |
|  | **N** | *n* | *r* | *b* | *b* | *b* | *b* | *b* | *d* | *b* | *b* |
|  | **Cr** | *b* | *s* | *p* | *s* | *i* | *b* | *b* | *b* | *d* | *b* |
|  | **Ar** | *b* | *s* | *s* | *p* | *b* | *i* | *b* | *b* | *b* | *d* |
|  | **Gr** | *r* | *p* | *s* | *s* | *b* | *b* | *i* | *d* | *b* | *b* |
|  | **ICr** | *b* | *s* | *p* | *s* | *i* | *b* | *b* | *b* | *d* | *b* |
|  | **IAr** | *b* | *s* | *s* | *p* | *b* | *i* | *b* | *b* | *b* | *d* |
|  | **IGr** | *r* | *p* | *s* | *s* | *b* | *b* | *i* | *d* | *b* | *b* |
|  | **DCr** | *b* | *s* | *s* | *p* | *b* | *i* | *b* | *b* | *b* | *d* |
|  | **DAr** | *r* | *p* | *s* | *s* | *b* | *b* | *i* | *d* | *b* | *b* |
|  | **DGr** | *b* | *s* | *p* | *s* | *i* | *b* | *b* | *b* | *d* | *b* |

Table S3. An example of starting probability of 10 hidden states in HMM for a trinucleotide repeat. ‘*n*’, ‘*r*’, ‘*d*’ and ‘*b*’ indicated the initial probability of non-repeat, the first repeat state, the deletion rate of the first repeat state and background random error. In this study, ‘*b* was set to absolute minimum larger than 0, ‘*n*’=0.96, ‘*r*’=0.02 and ‘*d*’=0.02.

| **N** | **Cr** | **Ar** | **Gr** | **ICr** | **IAr** | **IGr** | **DCr** | **DAr** | **DGr** |
| --- | --- | --- | --- | --- | --- | --- | --- | --- | --- |
| *n* | *r* | *b* | *b* | *b* | *b* | *b* | *d* | *b* | *b* |

Table S4. The statistics of raw reads and CCS reads for 20 patients with SCA3 and 5 control subjects. N50 referred to the length for which the collection of all sequences of that length or longer contained at least half of the sum of the lengths of all sequences.

|  | **raw reads** | | | | **CCS reads** | | | |
| --- | --- | --- | --- | --- | --- | --- | --- | --- |
| ***Subject*** | ***Num of reads*** | ***Total bases of reads*** | ***N50 of subreads*** | ***Mean of subreads*** | ***Num of reads*** | ***Total bases of reads*** | ***N50 of subreads*** | ***Mean of subreads*** |
| All | 585,646 | 939,895,440 | 1,672 | 1,658 | 38,058 | 61,063,678 | 1,590 | 1,589 |
| sam001 | 28,417 | 44,845,533 | 1,632 | 1,621 | 1,804 | 2,848,432 | 1,549 | 1,549 |
| sam002 | 29,485 | 47,747,157 | 1,688 | 1,676 | 1,913 | 3,104,261 | 1,599 | 1,599 |
| sam003 | 23,846 | 37,534,156 | 1,634 | 1,623 | 1,547 | 2,431,518 | 1,549 | 1,549 |
| sam004 | 16,988 | 26,753,750 | 1,635 | 1,624 | 1,086 | 1,707,598 | 1,549 | 1,549 |
| sam005 | 29,157 | 46,235,489 | 1,649 | 1,639 | 1,859 | 2,953,339 | 1,567 | 1,567 |
| sam006 | 28,057 | 44,112,774 | 1,629 | 1,619 | 1,761 | 2,766,671 | 1,548 | 1,548 |
| sam007 | 25,612 | 41,189,825 | 1,672 | 1,661 | 1,659 | 2,670,766 | 1,587 | 1,587 |
| sam008 | 25,293 | 39,937,435 | 1,637 | 1,625 | 1,602 | 2,527,421 | 1,549 | 1,549 |
| sam009 | 28,669 | 46,532,994 | 1,686 | 1,674 | 1,851 | 3,005,244 | 1,596 | 1,596 |
| sam010 | 25,650 | 41,324,099 | 1,671 | 1,660 | 1,673 | 2,694,537 | 1,587 | 1,587 |
| sam011 | 21,296 | 34,943,290 | 1,704 | 1,690 | 1,391 | 2,288,891 | 1,612 | 1,612 |
| sam012 | 23,629 | 38,309,517 | 1,679 | 1,667 | 1,533 | 2,490,092 | 1,593 | 1,593 |
| sam013 | 23,140 | 39,475,610 | 1,781 | 1,773 | 1,598 | 2,724,066 | 1,704 | 1,704 |
| sam014 | 24,681 | 39,735,867 | 1,672 | 1,661 | 1,592 | 2,561,893 | 1,587 | 1,587 |
| sam015 | 31,441 | 49,308,925 | 1,627 | 1,618 | 2,013 | 3,154,229 | 1,548 | 1,548 |
| sam016 | 26,475 | 43,915,369 | 1,736 | 1,721 | 1,774 | 2,948,539 | 1,636 | 1,636 |
| sam017 | 23,102 | 37,928,109 | 1,718 | 1,703 | 1,547 | 2,550,365 | 1,623 | 1,622 |
| sam018 | 22,464 | 36,317,920 | 1,687 | 1,678 | 1,439 | 2,320,398 | 1,615 | 1,614 |
| sam019 | 22,936 | 36,361,232 | 1,636 | 1,624 | 1,487 | 2,348,602 | 1,549 | 1,549 |
| sam020 | 22,238 | 35,199,126 | 1,637 | 1,625 | 1,441 | 2,273,444 | 1,549 | 1,549 |
| sam021 | 7,750 | 12,290,200 | 1,659 | 1,643 | 569 | 884,165 | 1,552 | 1,552 |
| sam022 | 27,510 | 44,086,964 | 1,674 | 1,666 | 1,777 | 2,846,215 | 1,602 | 1,602 |
| sam023 | 30,013 | 47,847,003 | 1,664 | 1,656 | 1,920 | 3,059,890 | 1,592 | 1,592 |
| sam024 | 6,915 | 10,933,074 | 1,666 | 1,648 | 504 | 783,633 | 1,552 | 1,552 |
| sam025 | 10,882 | 17,030,022 | 1,632 | 1,624 | 718 | 1,119,469 | 1,561 | 1,561 |

Table S5. The estimation of repeat counts for 20 patients with SCA3 and 5 control subjects from raw reads and CCS reads by RepeatHMM and TRhist. “al1” indicated smaller repeat count among two alleles, while “al2” indicated larger repeat count. “dif1” and “dif2” provided the difference of the estimated count minus the true count.

|  | **True counts** | | **Prediction**  **on raw reads** | | | | **Prediction**  **on CCS reads** | | | | **Prediction by TRhist**  **on raw reads** | | | |
| --- | --- | --- | --- | --- | --- | --- | --- | --- | --- | --- | --- | --- | --- | --- |
| ***Subject*** | ***al1*** | ***al2*** | ***al1*** | ***al2*** | ***dif1*** | ***dif2*** | ***al1*** | ***al2*** | ***dif1*** | ***dif2*** | ***al1*** | ***al2*** | ***dif1*** | ***dif2*** |
| sam001 | 14 | 77 | 14 | 78 | 0 | 1 | 14 | 83 | 0 | 6 | 5 | 47 | -9 | -30 |
| sam002 | 30 | 66 | 30 | 66 | 0 | 0 | 31 | 71 | 1 | 5 | 11 | 11 | -19 | -55 |
| sam003 | 14 | 69 | 14 | 70 | 0 | 1 | 14 | 74 | 0 | 5 | 5 | 14 | -9 | -55 |
| sam004 | 14 | 71 | 14 | 71 | 0 | 0 | 14 | 76 | 0 | 5 | 5 | 37 | -9 | -34 |
| sam005 | 21 | 72 | 20 | 72 | -1 | 0 | 20 | 77 | -1 | 5 | 7 | 47 | -14 | -25 |
| sam006 | 14 | 77 | 14 | 78 | 0 | 1 | 14 | 83 | 0 | 6 | 5 | 14 | -9 | -63 |
| sam007 | 26 | 71 | 26 | 72 | 0 | 1 | 27 | 77 | 1 | 6 | 9 | 47 | -17 | -24 |
| sam008 | 14 | 63 | 14 | 64 | 0 | 1 | 14 | 68 | 0 | 5 | 5 | 14 | -9 | -49 |
| sam009 | 29 | 70 | 29 | 70 | 0 | 0 | 29 | 75 | 0 | 5 | 11 | 48 | -18 | -22 |
| sam010 | 27 | 71 | 26 | 73 | -1 | 2 | 27 | 77 | 0 | 6 | 10 | 50 | -17 | -21 |
| sam011 | 34 | 75 | 33 | 77 | -1 | 2 | 35 | 82 | 1 | 7 | 12 | 12 | -22 | -63 |
| sam012 | 28 | 89 | 28 | 90 | 0 | 1 | 28 | 96 | 0 | 7 | 10 | 46 | -18 | -43 |
| sam013 | 61 | 61 | 61 | 62 | 0 | 1 | 64 | 67 | 3 | 6 | 17 | 17 | -44 | -44 |
| sam014 | 26 | 65 | 26 | 66 | 0 | 1 | 27 | 69 | 1 | 4 | 9 | 63 | -17 | -2 |
| sam015 | 14 | 89 | 14 | 91 | 0 | 2 | 14 | 96 | 0 | 7 | 5 | 14 | -9 | -75 |
| sam016 | 40 | 67 | 40 | 68 | 0 | 1 | 42 | 72 | 2 | 5 | 13 | 13 | -27 | -54 |
| sam017 | 37 | 68 | 37 | 69 | 0 | 1 | 38 | 74 | 1 | 6 | 12 | 12 | -25 | -56 |
| sam018 | 28 | 40 | 28 | 40 | 0 | 0 | 29 | 42 | 1 | 2 | 10 | 10 | -18 | -30 |
| sam019 | 14 | 82 | 14 | 85 | 0 | 3 | 14 | 90 | 0 | 8 | 5 | 43 | -9 | -39 |
| sam020 | 14 | 68 | 14 | 70 | 0 | 2 | 14 | 74 | 0 | 6 | 5 | 36 | -9 | -32 |
| sam021 | 14 | 14 | 14 | 14 | 0 | 0 | 14 | 14 | 0 | 0 | 5 | 10 | -9 | -4 |
| sam022 | 27 | 35 | 27 | 35 | 0 | 0 | 27 | 36 | 0 | 1 | 9 | 13 | -18 | -22 |
| sam023 | 28 | 28 | 28 | 28 | 0 | 0 | 28 | 29 | 0 | 1 | 9 | 9 | -19 | -19 |
| sam024 | 14 | 14 | 14 | 14 | 0 | 0 | 14 | 14 | 0 | 0 | 5 | 10 | -9 | -4 |
| sam025 | 14 | 19 | 14 | 19 | 0 | 0 | 14 | 19 | 0 | 0 | 5 | 14 | -9 | -5 |

Table S6. The estimation of repeat counts for 20 patients with SCA3 and 5 control subjects on raw reads and CCS reads by BAMself. “al1” indicated smaller repeat count among two alleles, while “al2” indicated larger repeat count. “dif1” and “dif2” provided the difference of the estimated count minus the true count.

|  | **True counts** | | **Prediction**  **on raw reads** | | | | **Prediction**  **on CCS reads** | | | |
| --- | --- | --- | --- | --- | --- | --- | --- | --- | --- | --- |
| ***subject*** | ***al1*** | ***al2*** | ***al1*** | ***al2*** | ***dif1*** | ***dif2*** | ***al1*** | ***al2*** | ***dif1*** | ***dif2*** |
| sam001 | 14 | 77 | 15 | 15 | 1 | -62 | 14 | 14 | 0 | -63 |
| sam002 | 30 | 66 | 31 | 31 | 1 | -35 | 31 | 31 | 1 | -35 |
| sam003 | 14 | 69 | 15 | 15 | 1 | -54 | 14 | 14 | 0 | -55 |
| sam004 | 14 | 71 | 15 | 15 | 1 | -56 | 14 | 14 | 0 | -57 |
| sam005 | 21 | 72 | 21 | 21 | 0 | -51 | 20 | 20 | -1 | -52 |
| sam006 | 14 | 77 | 15 | 15 | 1 | -62 | 14 | 14 | 0 | -63 |
| sam007 | 26 | 71 | 27 | 27 | 1 | -44 | 26 | 26 | 0 | -45 |
| sam008 | 14 | 63 | 15 | 15 | 1 | -48 | 14 | 14 | 0 | -49 |
| sam009 | 29 | 70 | 31 | 31 | 2 | -39 | 30 | 30 | 1 | -40 |
| sam010 | 27 | 71 | 27 | 27 | 0 | -44 | 26 | 26 | -1 | -45 |
| sam011 | 34 | 75 | 15 | 36 | -19 | -39 | 35 | 35 | 1 | -40 |
| sam012 | 28 | 89 | 29 | 29 | 1 | -60 | 28 | 28 | 0 | -61 |
| sam013 | 61 | 61 | 15 | 15 | -46 | -46 | 14 | 14 | -47 | -47 |
| sam014 | 26 | 65 | 27 | 27 | 1 | -38 | 26 | 26 | 0 | -39 |
| sam015 | 14 | 89 | 15 | 15 | 1 | -74 | 14 | 14 | 0 | -75 |
| sam016 | 40 | 67 | 15 | 43 | -25 | -24 | 42 | 42 | 2 | -25 |
| sam017 | 37 | 68 | 15 | 39 | -22 | -29 | 38 | 38 | 1 | -30 |
| sam018 | 28 | 40 | 30 | 43 | 2 | 3 | 29 | 42 | 1 | 2 |
| sam019 | 14 | 82 | 15 | 15 | 1 | -67 | 14 | 14 | 0 | -68 |
| sam020 | 14 | 68 | 15 | 15 | 1 | -53 | 14 | 14 | 0 | -54 |
| sam021 | 14 | 14 | 15 | 15 | 1 | 1 | 14 | 14 | 0 | 0 |
| sam022 | 27 | 35 | 28 | 37 | 1 | 2 | 27 | 36 | 0 | 1 |
| sam023 | 28 | 28 | 29 | 29 | 1 | 1 | 28 | 28 | 0 | 0 |
| sam024 | 14 | 14 | 15 | 15 | 1 | 1 | 14 | 14 | 0 | 0 |
| sam025 | 14 | 19 | 15 | 20 | 1 | 1 | 14 | 19 | 0 | 0 |

Table S7 The estimation of 15 types of trinucleotide repeats on HX1 and NA12878 by RepeatHMM. ‘Normal’ and ‘Pathogenic’ indicated the known range of the normal and pathogenic repeats, respectively. “NA” (for NA12878 only) indicated that there was no enough coverage to infer repeat counts for the corresponding dataset.

| Gene | Normal | Pathogenic | HX1 (PacBio) | NA12878 (Illumina) | NA12878 (PacBio) | NA12878 (Nanopore) | Disorder |
| --- | --- | --- | --- | --- | --- | --- | --- |
| *aff2* | 6 to 35 | 200+ | 36,36 | 28,28 | 23,23 | 24,24 | fragile XE mental retardation |
| *ar* | 9 to 36 | 38 to 62 | 22,22 | 20,24 | NA,NA | 28,44 | spinal and bulbar muscular atrophy |
| *atn1* | 6 to 35 | 49 to 88 | 14,14 | 19,19 | 17,19 | 18,18 | dentatorubropallidoluysian atrophy |
| *atxn1* | 6 to 35 | 49 to 88 | 28,28 | 29,30 | 29,29 | 26,29 | spinocerebellar ataxia type 1 |
| *atxn2* | 14 to 32 | 33 to 77 | 21,21 | 22,23 | 22,22 | 21,21 | spinocerebellar ataxia type 2 |
| *atxn3* | 12 to 40 | 55 to 86 | 14,14 | 23,24 | 23,23 | 22,22 | spinocerebellar ataxia type 3 |
| *atxn7* | 7 to 17 | 38 to 120 | 10,10 | 10,12 | 9,12 | 9,9 | spinocerebellar ataxia type 7 |
| *atxn8os* | 16 to 37 | 110 to 250 | 9,17 | 15,16 | 15,15 | 14,14 | spinocerebellar ataxia Type 8 |
| *cacna1a* | 4 to 18 | 21 to 30 | 13,13 | 11,12 | 10,12 | 11,11 | spinocerebellar ataxia type 6 |
| *dmpk* | 5 to 37 | 50+ | 11,14 | 5,13 | 5,13 | NA, NA | myotonic dystrophy |
| *fmr1* | 6 to 53 | 230+ | 32,34 | 30,31 | NA,NA | NA, NA | fragile X syndrome |
|  | 6 to 53 | 55-200 |  |  |  |  | fragile X-associated tremor/ataxia syndrome |
| *fxn* | 7 to 34 | 100+ | 9,9 | 9,10 | 8,11 | 7,7 | Friedreich's ataxia |
| *htt* | 6 to 35 | 36 to 250 | 17,17 | 18,20 | 19,19 | 14,19 | Huntington's disease |
| *ppp2r2b* | 7 to 28 | 66 to 78 | 10,10 | 10,14 | 9,NA | 14,14 | spinocerebellar ataxia type 12 |
| *tbp* | 25 to 42 | 47 to 63 | 37,37 | NA,NA | 39,39 | 35,39 | spinocerebellar ataxia type 17 |


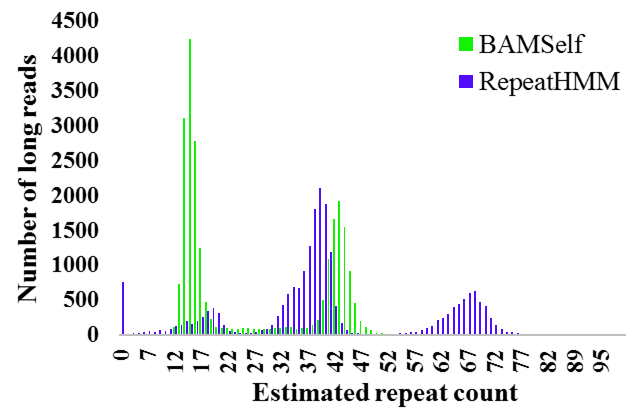


Figure S1. An example demonstrating the failure of alignment-based approach BAMself (analysis of each alignment of those long reads which fully covered the repeat region and its flanking sequences in the aligned BAM file). The subject of interest had 40 and 67 CTG repeats for two alleles in the *ATXN3* gene, respectively. The estimation by BAMSelf was 15 and 43, while the estimation by RepeatHMM was 40 and 68.


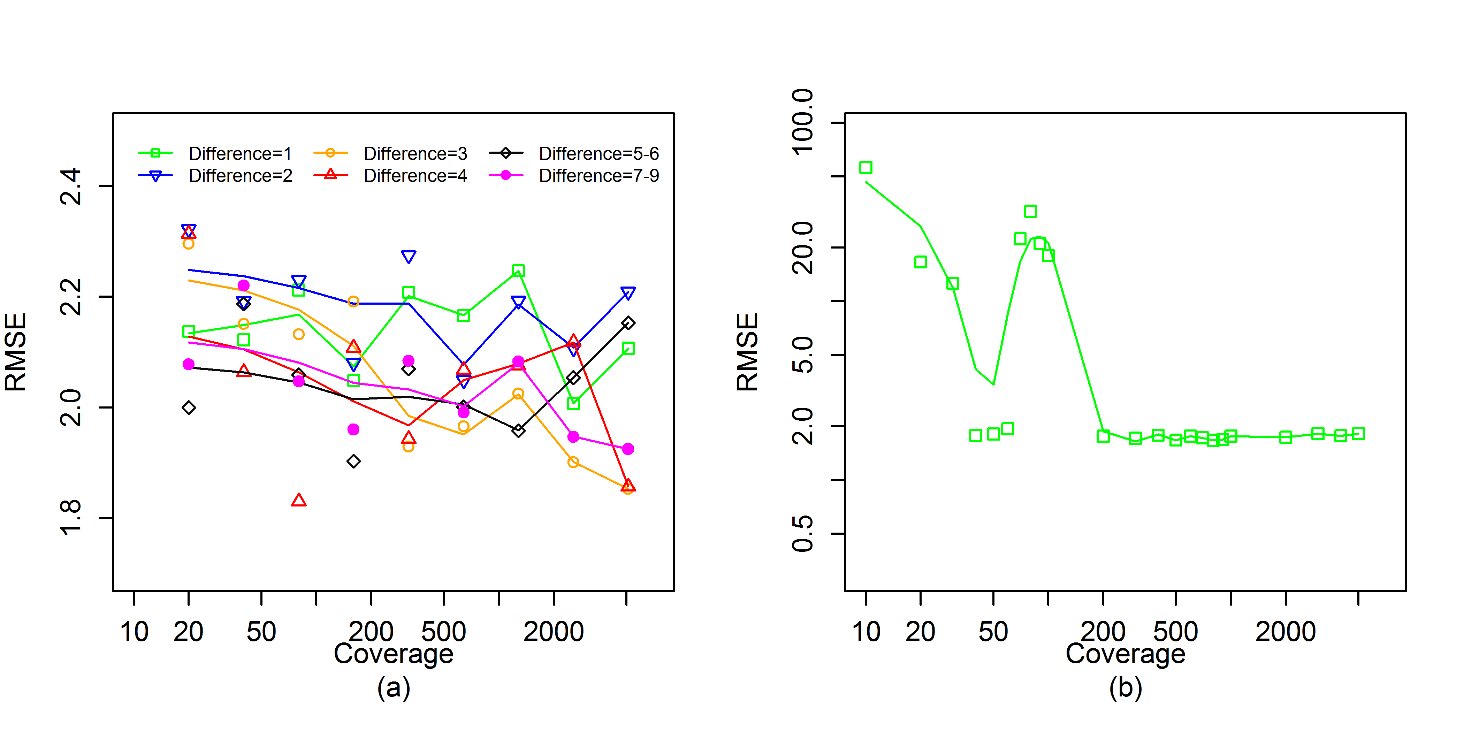


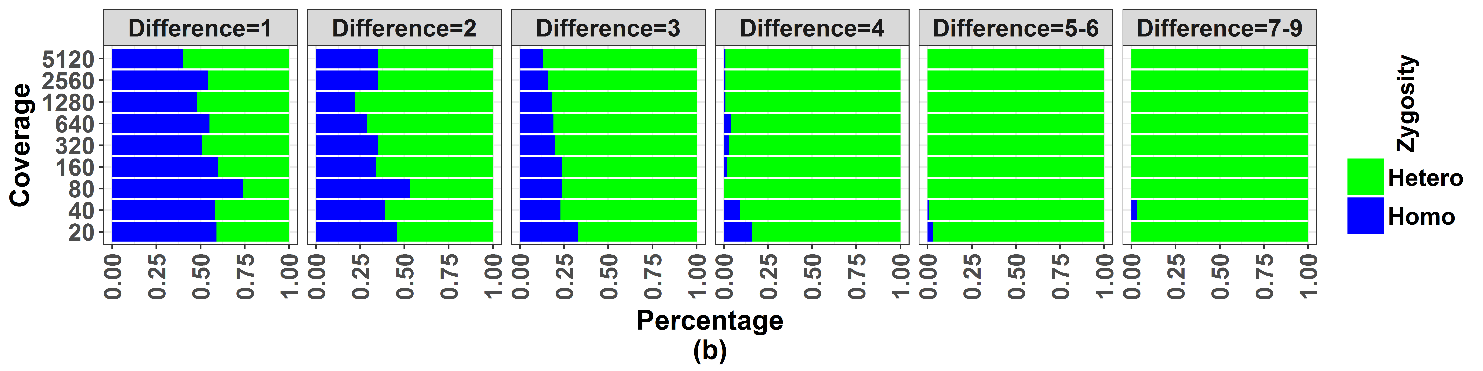


Figure S2 (a) The performance of RepeatHMM for two alleles with similar repeat counts. The difference of two alleles varied from 1, 2, 3, 4, 5 to 6, and 7 to 9 in the simulation. (b) The percentage of homozygous calls for alleles with similar repeat counts. ‘Hetero’ indicated heterozygous calls, and ‘Homo’ indicated homozygous calls. RepeatHMM tended to call heterozygous variants as homozygous when the differences between two alleles was less than 3.
